# Adherence to the EAT-Lancet diet and risk of colorectal cancer in the general population and among individuals with diabetes: a cohort study

**DOI:** 10.1007/s00394-026-03936-6

**Published:** 2026-03-09

**Authors:** Line B. Rosendal, Charlotte Bundgaard, Jie Zhang, Anja Olsen, Agnetha L. Rostgaard-Hansen, Christina C. Dahm, Daniel B. Ibsen

**Affiliations:** 1 Department of Public Health, Aarhus University, Bartholins Allé 2, Aarhus, Denmark; 2 Steno Diabetes Center Aarhus, Aarhus University Hospital, Aarhus, Denmark; 3 Danish Cancer Institute, Diet, Cancer and Health, Copenhagen, Denmark

**Keywords:** Cohort study, EAT-Lancet diet, Colorectal cancer, Sustainable diets, Epidemiology

## Abstract

**Background:**

Healthy and sustainable diets, such as the EAT-Lancet diet, may benefit planetary and human health, though evidence for colorectal cancer (CRC) is limited. This study examined the association between EAT-Lancet diet adherence and CRC risk in middle-aged Danes, including subgroup analysis among individuals with diabetes.

**Methods:**

Based on data from the Danish Diet, Cancer, and Health cohort (1993–1997), we included 55,651 participants aged 50–64 without cancer at baseline. Adherence to the EAT-Lancet diet was evaluated using a diet score (0–42 points, 42 indicating highest adherence) from a validated food frequency questionnaire. Cox proportional hazard models were used to estimate hazard ratios (HR) for CRC, colon cancer, and rectal cancer. The pseudo-observation method was used to estimate risk differences after 20 years.

**Results:**

In total 1877 participants were diagnosed with CRC (median follow-up: 18.7 years). Multivariable-adjusted HRs for CRC, colon cancer, and rectal cancer were 0.75 (95% CI: 0.60, 0.93), 0.73 (95% CI: 0.57, 0.95), and 0.76 (95% CI: 0.50, 1.16) for highest (24–34 points) versus lowest adherence (9–16 points), respectively. The 20-year RD for CRC was -0.60% (95% CI: -1.27, 0.06).

**Conclusions:**

Higher adherence to the EAT-Lancet diet was associated with lower risk of CRC in middle-aged Danes.

**Electronic supplementary material:**

The online version of this article (10.1007/s00394-026-03936-6) contains supplementary material, which is available to authorized users.

## Background

Cancer is the leading cause of death worldwide, with nearly 10 million attributable deaths in 2020. Colorectal cancer (CRC) is the third most common cancer worldwide, with more than 1.9 million new cases in 2020 (1), (2). Several studies have investigated the association between healthy lifestyles and the risk of CRC and have found a significantly lower CRC risk when following a diet high in vegetables, fruits, whole grains, olive oil, fish, soy, poultry, and low-fat dairy, and a low intake of red meat and processed foods (3). A diet high in antioxidants, dietary fibre, selenium, carotenoids, and minerals has been proposed to lower the risk of developing CRC through various mechanisms, such as promoting short-chain fatty acid production, lowering exposure to carcinogenic compounds and minimizing the risk of hyperinsulinemia (4), (5), (6).

An emerging risk factor for development of cancer is the presence of diabetes, and cancer is one of the leading contributors to death in people with diabetes (7), (8). The prevalence of diabetes is increasing and has reached half a billion worldwide and is predicted to rise to 783 million people in 2045 (9). More than 75,000 new cancer cases each year are estimated to be attributed to diabetes, and people with diabetes have an elevated risk of CRC (10). The higher risk of developing cancer in people with diabetes may be caused by hyperinsulinemia, hyperglycaemia, or chronic inflammation (7). Besides, individuals with diabetes also have elevated levels of Insulin-like Growth Factor-1, which can affect the development of cancer indirectly (11–13). However, the increased risk of CRC in individuals with type 2 Diabetes may be modified by lifestyle factors such as diet and physical activity (7, 10).

In 2019, the EAT-Lancet Commission on healthy diets from sustainable food systems presented a reference diet called the EAT-Lancet diet as a healthy environmentally sustainable diet, focusing on preventing diet-related chronic diseases and mortality (14). The EAT-Lancet diet mainly consists of vegetables and fruits, whole grains, legumes, nuts, and unsaturated oils. Furthermore, it consists of low to moderate consumption of fish, shellfish and poultry, and zero to low consumption of red meat, processed meat, added sugars, refined grains, and starchy vegetables (14). The EAT-Lancet Commission aims, with the reference diet, to reduce environmental degradation due to food production, and to contribute significant support to increase health among the world's population (14).

Few previous studies have investigated the association between adherence to the EAT-Lancet diet and risk of CRC. One study found that higher adherence compared to lower adherence is associated with lower risk of CRC (15). However, the two other studies found no significant association, thereby necessitating further research (16, 17). Those studies were performed in healthy individuals, and research examining the association between adherence to the EAT-Lancet diet and the risk of CRC in people with diabetes is sparse. We investigated the association between adherence to the EAT-Lancet diet and risk of CRC, including colon cancer (CC) and rectal cancer (RC) in middle-aged adults, and in people with diabetes at baseline. We hypothesised that both individuals with and without diabetes at baseline who had high adherence to the EAT-Lancet diet had lower risk of CRC.

## Methods

### Study design and study population

This study was based on data from the Danish Diet, Cancer, and Health cohort (DCH). The DCH cohort has been described in detail elsewhere (18). Overall, 160,725 individuals were invited to participate if they lived in Copenhagen or Aarhus County, if they were between 50–64 years, and if they were not diagnosed with cancer registered in the Danish Cancer Registry (19). The initial data collection took place in 1993–1997 and resulted in a cohort of 57,053 participants. Participants completed questionnaires on diet and lifestyle, which were optically scanned at the study centre to check for errors and missing information. The self-administered questionnaire was developed specifically for the DCH cohort (19). Anthropometric and other biological measurements from participants were gathered at two study centres (19).

**Fig. 1:**
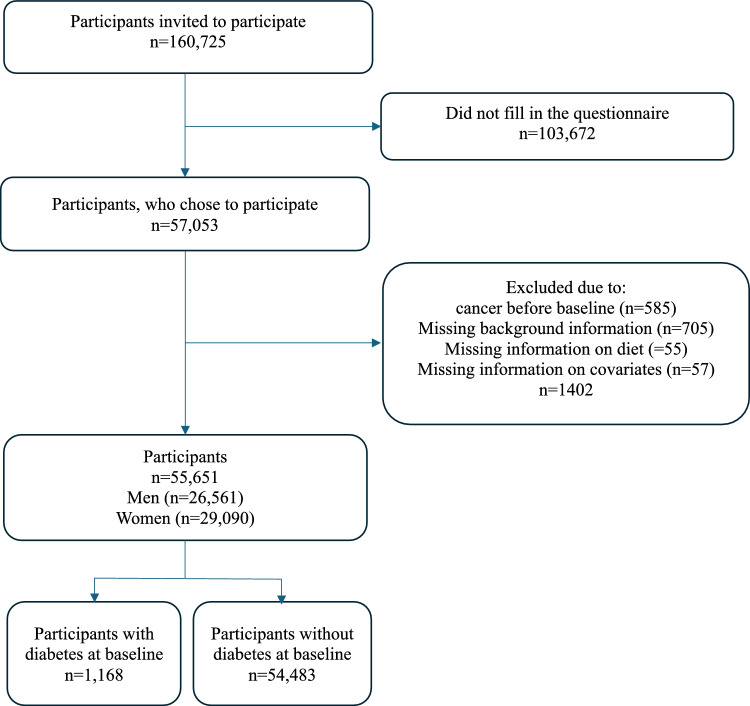
Flow chart of the study population from the danish, diet, cancer, and health study eligible for statistical analysis in the present study. Participants included had no prior diagnosis of cancer at baseline and provided complete information on all covariates

Participants with incomplete or missing data on diet, background information, and potential confounders were excluded in the present study (Fig. 1).

In secondary analyses, the association between adherence to the EAT-Lancet diet and CRC in participants with diabetes at baseline was examined. Diabetes at baseline was defined if self-reported in the lifestyle questionnaire at baseline. The participants could answer ‘yes’, ‘no’, and ‘do not know’ if they had diabetes. Participants who answered that they did not know if they had diabetes and participants who answered that they did not have diabetes were treated as a separate group.

### EAT-Lancet diet index

Information on diet was collected at baseline with a food frequency questionnaire (FFQ), which was sent by letter. The FFQ gathered information on participants’ food and beverage intake over the past 12 months and contained 192 items in 12 categories ranging from never to more than eight times a day (19). The questionnaire was checked by interviewers and intake of specific foods and nutrients was calculated by the software program FoodCalc, which used specifically developed standardised recipes and portions sizes (18). The FFQ has been validated for assessment of food, energy, and nutrient intake and was compared with two seven-day diaries, where the subjects were instructed to describe all food items in as much detail as possible, and the food was weighed (19).

Adherence to the EAT-Lancet diet was based on participants’ average intake from the FFQ. The diet score used in this study was developed by Stubbendorff et al. (20). It consists of 14 components and points were given between 0–3 for each component in relation to the level of adherence. A score of 0 was given for intakes far from the recommended levels, while 1 and 2 represented partial adherence. A score of 3 was given when intake was within or above (for beneficial components) or below (for limited components) the recommended range. The components consist of whole grains, potatoes, vegetables, fruits, dairy, beef and lamb, pork, chicken, eggs, fish, legumes, nuts, unsaturated oils, and added sugar (Supplementary Table 1). It is possible to score between 0–42 points, where 0 is lowest adherence and 42 is highest adherence. The diet index was based on the EAT-Lancet reference diet (14).

### Colorectal cancer

The outcome was diagnosis of CRC during the follow-up period, analysed both as a combined outcome and divided into CC and RC. Information on cancer diagnoses from the DCH study was obtained through register linkage to the Danish Cancer Registry using the unique personal identification number assigned to every Danish citizen at birth. Cancer was defined using International Classification of Diseases 10 codes: C18 (colon cancer), C19 (cancer in the rectosigmoid junction) and C20 (rectal cancer) (21, 22).

### Covariates

Information on potential confounders was collected via the self-administered lifestyle questionnaire at baseline and the FFQ. Potential confounders selected a priori based on previous literature and related to the EAT-Lancet diet and CRC were: age, sex, BMI, smoking status, physical activity level, alcohol consumption, education level, diabetes, energy intake, hypercholesterolemia and hypertension. Smoking status was categorised as ‘Never smoked’, ‘former smoker', ‘smoker 1–14 g tobacco/day’, ‘smoker 15–24 g tobacco/day’ and ‘smoker ≥ 25 g tobacco/day’. Physical activity level was dichotomised into less than 30 min of physical activity daily and at least 30 min of physical activity daily, based on recommendation from the Danish National Board of Health for adults (23). Education level was assessed in four categories: ‘vocational education (e.g., hairdresser or plumber), ‘short education’ (e.g., only primary school, high school or one or more short courses), ‘medium education’ (e.g., social worker or BSc degree) and ‘long education (e.g., MSc or higher degree from university). Information on alcohol and energy intake was assessed through the FFQ. Participants could answer ‘yes’, ‘no’, and ‘do not know’ to whether they had a history of hypertension and/or a history of hypercholesterolaemia. Participant height and weight were collected during the baseline visit to a study center by trained professionals. Height was measured to the nearest 0.5 cm without shoes, and body weight was measured with a digital scale, and recorded to the nearest 100 g, with the participant wearing light underwear. BMI was calculated as weight divided by height squared (kg/m^2) (19).

### Statistical analysis

Descriptive statistics for baseline characteristics were presented for all participants and across groups of adherences to the EAT-Lancet diet. Cox proportional hazards models, with age as the underlying timescale, were used to calculate hazard ratios (HR) with 95% confidence intervals (95% CI) for the development of CRC. Participants were considered at risk of developing cancer from their age at recruitment until age at diagnosis with CRC, loss to follow-up, change in personal identification number, emigration, death of other causes than CRC, or administrative censoring on December 31st, 2016, whichever came first.

The EAT-Lancet diet score was analysed as a categorical variable in five groups by dividing the distribution of EAT-Lancet diet score in the study population into approximate quintiles to ensure a balanced number of participants in each category (≤ 16 points, 17–19 points, 20–21 points, 22–23 points, and ≥ 24 points). It was also analysed as a restricted cubic spline variable, with knots automatically placed at percentiles dividing the distribution into four equal parts (16, 19, 21, and 25 points). The group of participants with the lowest diet index score was the reference group for the categorical analyses, while the reference for the cubic spline analysis was set at 10 points. The analysis was performed in four models. Model 1a was adjusted for age and sex and as strata-variables tertiles of enrolment date; Model 1b was further adjusted for diabetes, smoking status, physical activity level, alcohol consumption, history of hypercholesterolemia, history of hypertension and educational level; Model 2 was further adjusted for BMI and Model 3 was further adjusted for energy intake. Both overall incidence of CRC and subtypes, CC and RC were investigated. In the analysis of CC, participants who developed RC were censored at the time of their RC diagnosis, as only the first CRC diagnosis was considered. Similarly, in the analysis of RC, participants who developed CC were censored at the time of their CC diagnosis. The proportional hazards assumption was checked with a log-minus-log survival curve. The assumptions of independence between entry time and the outcome were checked and satisfied when stratifying entry dates into tertiles.

We analysed data on the additive scale using the pseudo-observational method with death as a competing event and calendar time as the time scale. The assumptions of independent entry time and censoring were checked and satisfied when we stratified pseudo-values into four separate strata based on quartiles of dates of enrolment as in a previous study (24). Because the pseudo-observations estimate the individual event status at a specified time point with no censoring, several time points were chosen in the analyses, including 5, 10, 15, and 20 years to investigate the patterns in associations over time. In the second step, we ran a generalised linear model of the pseudo-observations using the identity link function to estimate risk differences (RDs). Age was adjusted for in the models using a restricted cubic spline variable with five knots and additional confounders were included in model 2.

Because people with diabetes have a higher risk of developing CRC compared to healthy individuals, analyses were stratified according to diabetes status at baseline in a continuous analysis. Additionally, a categorical and continuous analysis was made on the entire study population, stratified by sex. All stratified analyses were supplemented with a test for interaction using a Z-test for the interaction term with the EAT-Lancet diet.

As a robustness check, we conducted a sensitivity analysis in which the EAT-Lancet diet was categorized into quartiles, ensuring approximately equal numbers of participants in each group. Hazard ratios for quartiles were estimated using the same Cox proportional Hazards models as in the main analysis. In another sensitivity analyses, we examined the risk of bias due to potential reverse causation by excluding the first 4 years of follow-up time. Additionally, a sensitivity analysis was performed to examine whether the estimates changed when including participants who answered ‘do not know’ if they have diabetes, in the analysis for participants with diabetes. The statistical significance was based on a two-sided* P*-value < 0.05. Programming was performed using Stata version 17.0.

## Results

### Description of study population

In total, 55,651 participants were included in the analysis, 1877 were diagnosed with CRC during a median follow-up of 18.7 years (Fig. 1.), 268 participants emigrated or changed their personal identification number during the follow-up period, and 12,316 died of other causes than CRC during follow-up. In total 1168 participant had diabetes at baseline and of these 51 were subsequently diagnosed with CRC.

The median age at baseline was 56 years (p10-90: 52–60). Table 1 shows the distribution of baseline characteristics on all participants as well as the distribution in the five groups based on their score. The participants in this study obtained between 9 and 34 points with a median EAT-Lancet diet score of 20 points (p10-90: 17–24). Overall, participants in the group with the highest adherence (quintile 5) were more likely to be women, to have more than 9 years of education, not to be current smokers and to perform more than 30 min of physical activity per day. Also, they had the lowest intake of alcohol and the lowest BMI,

**Table 1:** Baseline characteristics of the danish diet, cancer, and health study participants included in the analysis of the association between EAT-Lancet diet score and risk of colorectal cancer

EAT-Lancet diet score	Total population n=55,651		<16 points n=5484 (9,9%)		17-19 points n=17,873 (32,1%)		20-21 points n=15,940 (28,6%)		22-23 points n=10,610 (19,1%)		>24 points n=5744 (10,3%)	
	Median p10-90		Median p10-90		Median p10-90		Median p10-90		Median p10-90		Median p10-90	
*Characteristics*												
Diet score points	20	17-24	16	14-16	18	17-19	20	20-21	22	22-23	25	24-27
Age, years	56	51-63	56	51-63	56	51-63	56	51-63	56	51-63	55	51-62
Alcohol intake (g/d)	12.9	1.6-47.4	14.5	1.6-64.4	14	2.0-55.2	13.5	2.0-45.2	13.3	2.0-44.2	11.6	1.6-38.3
Body mass index (kg/m^2)	25.54	21.4-31.1	26.2	21.7-31.9	25.8	21.7-31.4	25.5	21.5-31.1	25.3	21.3-30.8	24.6	20.8-30.2
Energy intake (kcal)	2117	1461-2954	2176	1486-3264	2142	1459-3089	2984	1486-2984	2102	1452-2881	2028	1430-2758
	n	%	n	%	n	%	n	%	n	%	n	%
Sex, men	26,561	48	3547	64,7	9775	54,7	7478	46,9	4148	39,1	1613	28,1
<9 years of education	8,251	15	1279	23,3	3172	17,7	2132	13,4	1142	10,8	526	9,2
Current smoker	20,041	36	2897	52,8	7447	41,7	5324	33,4	3016	28,4	1357	23,6
<30 min/day of physical activity	33,700	61	3865	70.5	11595	64.9	9455	59.3	5919	55.8	2866	49.9
Diabetes, yes	1,168	2	81	1.5	318	1.8	280	1.8	300	2.8	189	3.3
History of hypertension	9,050	16	833	15.2	2869	16.1	2651	16.6	1772	16.7	925	16.1
History of hypercholesterolaemia	4,164	7	346	6.3	1257	7.0	1192	7.5	875	8.2	494	8.6

All medians and percentiles are pseudo-medians percentiles as they represent at least an average of 5 surrounding values

### Association between adherence to the EAT-Lancet diet and risk of colorectal cancer

**Fig. 2:**
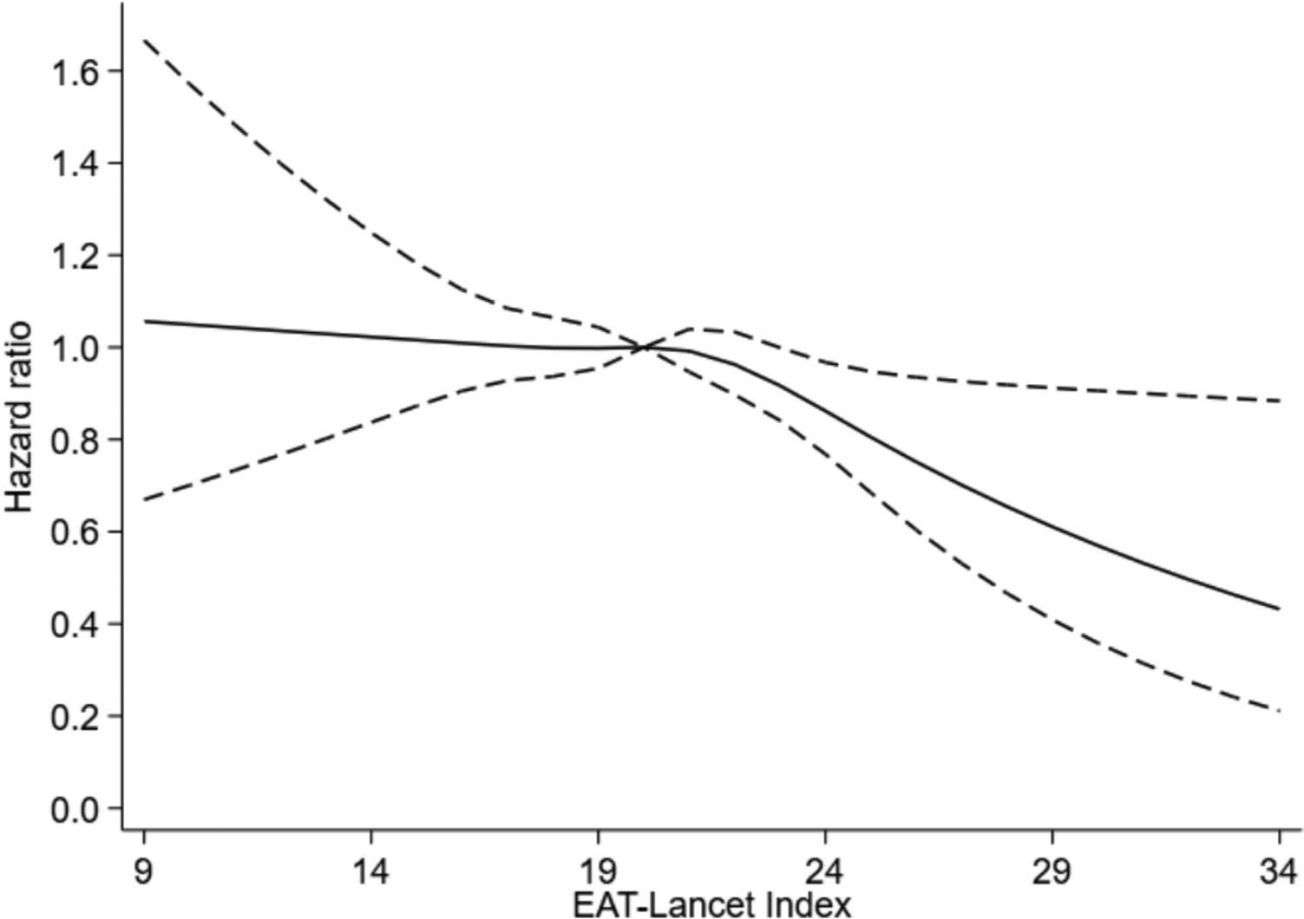
Graph of the association between EAT-Lancet diet score as restricted cubic splines and the risk of colorectal cancer. Bold line represents the fitted line, and the dashed line represents 95% CI

The risk of CRC was 27% lower (HR: 0.73; 95% CI: 0.59, 0.91) for the highest compared to the lowest adherence to the EAT-Lancet diet (Table 2, model 1b). Further adjusting for BMI (model 2) and energy intake (model 3) had minimal impact on the risk estimates (HR: 0.75; 95% CI: 0.60, 0.93, and HR: 0.74; 95% CI: 0.59, 0.93, respectively). A similar pattern was seen for the risk of CC but the results for RC were uncertain. Results from the analysis with the EAT-Lancet diet as a restricted cubic spline is presented in the graph below (Fig. 2), which shows that the HR for CRC was approximately stable with increasing EAT-Lancet diet score from 9 to 21 points, whereafter the HR for CRC decreased.

**Table 2:** HR and 95% CI for the association between adherence to the EAT-Lancet diet score and colorectal cancer, colon cancer and rectal cancer in the four adjusted models

HR for colorectal cancer	Events, *n*	Person-years	Model 1a*		Model 1b**		Model 2***		Model 3****	
EAT-Lancet diet score			HR	95% CI	HR	95% CI	HR	95% CI	HR	95% CI
< 16 points	204	97,386	Reference		Reference		Reference		Reference	
17–19 points	634	330,330	0.93	[0.80, 1.09]	0.95	[0.81, 1.11]	0.96	[0.82, 1.12]	0.96	[0.81, 1.12]
20–21 points	549	301,640	0.91	[0.77, 1.07]	0.94	[0.80, 1.11]	0.96	[0.81, 1.13]	0.95	[0.81, 1.12]
22–23 points	346	202,384	0.88	[0.73, 1.04]	0.91	[0.76, 1.09]	0.92	[0.77, 1.10]	0.92	[0.77, 1.10]
> 24 points	144	111,523	0.69	[0.55, 0.85]	0.73	[0.59, 0.91]	0.75	[0.60, 0.93]	0.74	[0.59, 0.93]
*HR for colon cancer*										
EAT-Lancet diet score										
< 16 points	146	97,386	Reference		Reference		Reference		Reference	
17–19 points	402	330,330	0.81	[0.67, 0.98]	0.83	[0.67, 1.01]	0.84	[0.96, 1.02]	0.84	[0.69, 1.01]
20–21 points	370	301,640	0.83	[0.69, 1.01]	0.87	[0.72, 1.06]	0.88	[0.73, 1.07]	0.88	[0.72, 1.07]
22–23 points	230	202,384	0.78	[0.64, 0.97]	0.82	[0.66, 1.02]	0.83	[0.67, 1.03]	0.83	[0.67, 1.03]
> 24 points	106	111,523	0.67	[0.52, 0.87]	0.72	[0.56, 0.93]	0.73	[0.57, 0.95]	0.73	[0.56, 0.95]
*HR for rectal cancer*										
*EAT-Lancet diet score*										
< 16 points	58	97,386	Reference		Reference		Reference		Reference	
17–19 points	232	330,330	1.23	[0.93, 1.65]	1.25	[0.93, 1.67]	1.26	[0.94, 1.68]	1.25	[0.94, 1.67]
20–21 points	179	301,640	1.10	[0.81, 1.45]	1.12	[0.83, 1.52]	1.14	[0.84, 1.54]	1.13	[0.84, 1.53]
22–23 points	116	202,384	1.12	[0.81, 1.52]	1.13	[0.82, 1.57]	1.15	[0.83, 1.59]	1.15	[0.83, 1.59]
> 24 points	38	111,523	0.71	[0.47, 1.08]	0.74	[0.49, 1.13]	0.76	[0.50, 1.16]	0.76	[0.50, 1.15]

All analyses were stratified by entry period (tertiles) in the Cox proportional hazard models

^*^Model 1a: Adjusted for age and sex

^**^ Model 1b: model 1a + diabetes, smoking status, physical activity level, alcohol consumption, education, hypertension, and hypercholesterolemia

^***^ Model 2: model 1b + BMI

^****^ Model 3: model 2 + total energy intake

When stratified for sex the Cox proportional hazard model indicated a lower risk of CRC (model 2, Supplementary Table 2) for the highest compared to the lowest adherence of a similar magnitude for both men and women (HR: 0.77; 95% CI: 0.56, 1.07 and HR: 0.77; 95% CI: 0.55–1.08), but the results were uncertain.

RDs were estimated using the pseudo-observation analysis for 5, 10, 15, and 20 years. The results indicated a lower risk of CRC for the highest compared to the lowest adherence, though with wide CIs. For example, at 20 years of follow-up the RD was -0.60% (95% CI: -1.27, 0.06) (Table 3).

**Table 3:** Cumulative risk differences (RD in percent) and 95% CI of CRC with adherence to the EAT-Lancet diet at 20 years

	5 years		10 years		15 years		20 years	
EAT-Lancet diet score	RD%	95% CI	RD%	95% CI	RD%	95% CI	RD%	95% CI
< 16 points	Reference		Reference		Reference		Reference	
17–19 points	0.02	[−0.02, 0.25]	0.05	[−0.01, 0.40]	0.06	[−0.39, 0.51]	−0.07	[−0.63, 0.50]
20–21 points	−0.01	[−0.34, 0.13]	−0.11	[−0.46, 0.24]	−0.01	[−0.47, 0.45]	−0.03	[−0.60, 0.55]
22–23 points	−0.06	[−0.31, 1.20]	−0.03	[−0.40, 0.35]	−0.02	[−0.51, 0.47]	−0.15	[−0.76, 0.47]
> 24 points	−0.16	[−0.44, 0.11]	−0.23	[−0.64, 0.17]	−0.40	[−0.93, 0.12]	−0.60	[−1.27, 0.06]

*RD risk difference, CRC colorectal cancer*

All analysis were adjusted for age, sex, diabetes, smoking status, physical activity level, alcohol consumption, education, hypertension, hypercholesterolemia and BMI

Among participants with pre-baseline diabetes, the results were too uncertain to make any conclusions (Supplementary Table 3).

### Sensitivity analysis

The sensitivity analysis using four equally sized groups showed a similar pattern as the main analysis, with the highest adherence group also having a lower risk of colorectal cancer. The HR were slightly attenuated, and the CI overlapped those from the main analysis. Thus, the results did not indicate any substantial changes in the direction or strength of the association, supporting the robustness of the main findings. Comparable results were observed for colon cancer, whereas the association for rectal cancer remained weaker and was not consistently statistically significant in any of the analyses (Supplementary Table 4). After excluding the first 4 years of follow-up time, 55,570 participants were included in the analysis, and 1796 were diagnosed with CRC during the remaining follow-up period. The results in Supplementary Table 5 shows numerically similar results to the analysis with all participants. After including participants who answered ‘do not know’ if they have diabetes in an analysis with participants who have diabetes, 3700 participants were included and 144 were diagnosed with CRC. The results in Supplementary Table 6 showed that the association between adherence to the EAT-Lancet diet and risk of CRC became less clear compared to the analysis which only included participants with certain diabetes. In addition, the CIs were wide.

## Discussion

In this cohort study, greater adherence to the EAT-lancet diet was associated with a lower risk of CRC. A similar association was shown for the risk of CC but the results for RC were uncertain. For participants with diabetes at baseline, the results were uncertain.

### Comparison with other studies

Our findings align with other large cohort studies that have investigated the association between adherence to the EAT-Lancet diet and CRC risk. A large French cohort study using data from the NutriNet-Santé cohort, found that the HR for CRC was 0.90 (95% CI: 0.58, 1.41) for the highest versus lowest adherence to the EAT-Lancet diet, indicating a lower risk, but with uncertain results (16). The EAT-Lancet scoring range in the French study was much wider (− 162 to 332) than in our study, which could suggest a greater variation in dietary patterns. However, differences in how adherence was scored may complicate direct comparisons. Additionally, they include refined grains in their scoring approach rather than whole grains, which might also contribute to the differences in the findings. A similar prospective study including 98,415 American adults found that higher adherence to the EAT-Lancet diet was associated with a lower CRC risk (HR: 0.81, 95% CI: 0.67, 0.98) for highest adherence versus lowest adherence, supporting the results from our study (15). In this study participants were divided into quartiles similar to our study ranging from ≤ 18, 19–21, 22–24, and ≥ 25 points. This may explain why their findings are consistent with ours. Additionally, an analysis in the UK Biobank study found that highest versus lowest adherence to the EAT-Lancet diet was linked to a lower risk of CRC (HR: 0.90; 95% CI: 0.78, 1.03) (17). They categorized adherence into three groups (low adherence: 0–4 points, moderate adherence: 5–7 points, and high adherence: 8–11 points). This range is not as wide as in our study and may potentially limit the study’s ability to detect stronger associations. Several other studies also point towards an overall lower risk of CRC when following different types of plant-based dietary patterns (25–28). However, evidence on adherence to the EAT-Lancet diet and CRC risk was sparse.

### Potential mechanisms

The beneficial association with greater adherence to the EAT-Lancet diet could be explained by a higher intake of dietary fibre, antioxidants. selenium, carotenoids, and minerals, which are found in the recommended foods of the EAT-Lancet diet. In particular, a high fibre intake contributes to gut health by stimulating the production of short-chain fatty acids, such as butyrate, which has anti-inflammatory properties and may play a role in reducing CRC risk. Recent findings suggest that fibre from whole grains is particularly beneficial, with higher intake being associated with a lower CRC risk. Additionally, individuals with higher fibre intake tend to consume less red and processed meat, which lowers exposure to potentially harmful compounds such as nitrosamines (6) (4). Furthermore, this has an impact on diabetes as well, which therefore can have a double positive effect (5). However, as we did not observe a difference in CRC risk among individuals with diabetes when we examined for effect modification, this could indicate that dietary influences on gut microbiota composition and function play a more significant role. The EAT-Lancet diet may create a competitive advantage for beneficial bacterial populations, which could contribute to CRC risk reduction. Further studies are needed due to our limited population of individuals with diabetes at baseline.

### Public health implications

To assess the relevance of this study in a public health context and to provide information on the prevention of CRC, a pseudo-observation analysis was performed with absolute measures of the risk at 5, 10, 15, and 20 years of follow-up. Across all time points, a trend towards a lower risk of CRC was observed for the group with the highest adherence compared to the lowest adherence, but with wide confidence intervals. Based on the RD of -0.60% within 20 years of follow-up, adherence to the EAT-Lancet diet, could prevent approximately 6 CRC cases per 1,000 individuals over 20 years. In a global perspective, this reduction could translate into the prevention of a substantial number of CRC cases. This aligns with findings from other studies that examines the benefit of plant-based and sustainable diets on other health outcomes, such as other types of cancers, cardiovascular diseases and overall mortality (1, 14). These findings emphasize the broader public health effects of following plant-based and sustainable dietary patterns.

### Strengths and limitations

This study has multiple strengths. It contains information on a large cohort (n = 55,651), with 1877 CRC cases, which provided sufficient power. Data regarding the outcome of CRC was obtained via registers, which minimised the risk of information bias. Only few participants were lost to follow-up (0.48%), which ensures a very low risk of selection. In a sensitivity analysis, we categorized the EAT-Lancet diet score into quartiles instead of quintiles as in the main analysis, The results were highly consistent with the primary findings, with overlapping confidence intervals and similar effect estimates. This suggests that the observed associations were not driven by the choice of categorization, supporting the robustness of our findings. In another sensitivity analysis we excluded participants diagnosed with CRC within the first four years of the study to account for reverse causation due to any recent dietary changes. The similar results observed in the sensitivity analysis and the main analysis suggest that the influence of reverse causation was unlikely. To account for potential misclassification of diabetes status, we made another sensitivity analysis where we added those who answered, ‘do not know’ if they have diabetes, to those who answered ‘yes’. We chose to focus on self-reported diabetes status rather than registry data because the diabetes register available in our dataset only reliably captures incident cases from 1995 onwards (29). The association between adherence to the EAT-Lancet diet and risk of CRC became less clear compared to the analysis which only included participants with definite diabetes, with much uncertainty. Due to the limited number of participants with diabetes status at baseline, only a continuous analysis was performed. There are also several limitations. The EAT-Lancet diet score was constructed based on self-reported information, which can lead to misclassification. Due to the follow-up design, any misclassification is most likely non-differential. For the group with the highest adherence compared with the lowest adherence, this would likely bias the estimated association toward the null. However, for the categories in between, the direction of bias could be in either direction. The participants only had a single self-administered FFQ at baseline, in which they reported eating habits over the last 12 months. In general, dietary intake is difficult to measure and no assessment tool seems to be able to measure dietary intake perfectly (30, 31). However, the FFQ was found to be a useful instrument for ranking individuals according to their intake of various food groups in population-based studies (19). The observed EAT-Lancet diet score in this study population ranged from 9–34 points, although it was possible to score between 0 and 42 points. It is possible that a greater reduction in CRC risk could be observed at higher scores than 34. Conversely, at lower scores than 9, an increased risk of CRC might be expected. However, this remains speculative and cannot be confirmed due to the underlying dietary patterns of our study population. In the analysis, four different models were used to adjust for several relevant confounders, but risk of residual confounding cannot be excluded.

## Conclusion

In this cohort study, high compared to low adherence to the EAT-Lancet diet was associated with a lower risk of CRC among middle-age Danish men and women. This was particularly the case for CC, though the results for RC were less clear. Among individuals with diabetes, there was no clear effect modification. More studies in people with diabetes are needed to disentangle the role of the EAT-Lancet diet in development of CRC in this high-risk group.

## Electronic supplementary material

Below is the link to the electronic supplementary material.

Supplementary file 1

## Data Availability

This study was based on data from the Diet, Cancer and Health Cohort and can be acquired upon reasonable request from the Danish Cancer Society (dchdata@cancer.dk).
