# Supplementary material for: Adherence to the EAT-Lancet diet and risk of colorectal cancer in the general population and among individuals with diabetes: a cohort study
Source: Eur J Nutr. 2026 Mar 9;65(3):90. doi: 10.1007/s00394-026-03936-6 (PMC12971822; doi:10.1007/s00394-026-03936-6)
Supplement: Supplementary file 1 [file 394_2026_3936_MOESM1_ESM.docx]

Supplementary information

**Adherence to the EAT-Lancet diet and risk of colorectal cancer: a cohort study**

Line B. Rosendal^1^, Charlotte Bundgaard^1^, Jie Zhang^1,2^, Anja Olsen^1,3^, Agnetha L. Rostgaard-Hansen^3^, Christina C. Dahm^1^, Daniel B. Ibsen^1,2^

^1^Department of Public Health, Aarhus University, Aarhus, Denmark

^2^Steno Diabetes Center Aarhus, Aarhus University Hospital, Aarhus, Denmark.

^3^Danish Cancer Institute, Diet, Cancer and Health, Copenhagen, Denmark.

Correspondence: Daniel B. Ibsen ([dbi@ph.au.dk](mailto:dbi@ph.au.dk))

Department of Public Health, Aarhus University, Bartholins Allé 2, DK-8000 Aarhus C, Denmark

Table legends

Supplementary Table 1. EAT-Lancet diet scoring criteria based on components from the Danish Diet, Cancer, and Health cohort

Supplementary Table 2. HR and 95% CI for the association between adherence to the EAT-Lancet diet score and colorectal cancer by adherence to the EAT-Lancet diet stratified by sex

Supplementary Table 3: HR and 95% CI for the association between adherence to the EAT-Lancet diet score and colorectal cancer by adherence to the EAT-Lancet diet stratified by diabetes

Supplementary Table 4. HR and 95% CI for the association between adherence to the EAT-Lancet diet score and colorectal cancer, colon cancer and rectal cancer across quartiles in the four adjusted models

Supplementary Table 5. HR and 95% CI for the association between adherence to the EAT-Lancet diet score and colorectal cancer in model 2, four years after baseline

Supplementary Table 6. HR and 95% CI for the association between adherence to the EAT-lancet diet score and colorectal cancer in people with diabetes and who answered ‘don't know’ if they have diabetes, in the four adjusted models.

**Supplementary table 1** EAT-Lancet diet scoring criteria based on components from the Danish Diet, Cancer, and Health cohort.

| Component | Reference intake (g/day)* | Criteria for score (0-3)** | Foods*** |
| --- | --- | --- | --- |
| Whole grains | 232 | 0 points = <58  1 point = 58–116  2 points = 116–232  3 points = >232 | Cereals, wholegrain |
| Potatoes | 50 | 0 points = >200  1 point = 100-200  2 points = 50-100  3 points = <50 | Potatoes  Potatoes, fatty |
| Vegetables | 300 | 0 points = <100  1 point = 100-200  2 points = 200-300  3 points = >300 | Leafy vegetables, fruiting vegetables, other root vegetables, cabbages mushrooms, onion, garlic, stalk vegetables, sprouts |
| Fruits | 200 | 0 points = <50  1 point = 50-100  2 points = 100-200  3 points = >200 | Citrus fruits  Other fruits |
| Dairy | 250 | 0 points = >1000  1 point = 500-1000  2 points = 250-500  3 points = <250 | Skimmed milk  Semi-skimmed milk  Whole fat milk  Buttermilk  Fermented dairy, low fat  Fermented dairy, whole fat  Butter  Cheese |
| Beef, lamb, and pork**** | 14 | 0 points = >56  2 points = 28-56  4 points = 14-28  6 points = <14 | Red meat  Processed meat |
|  |  |  |  |
| Poultry | 29 | 0 points = >116  1 point = 58-116  2 points = 29-58  3 points = <29 | Poultry |
| Eggs | 13 | 0 points = >50  1 point = 25-50  2 points = 13-25  3 points = <13 | Eggs |
| Fish | 28 | 0 points = <7  1 point = 7-14  2 points =14-28  3 points = >28 | Fish, lean, fresh  Fish, lean, processed  Fish, medium fat, fresh  Fish, medium fat, processed.  Fish, fatty, fresh  Fish, fatty, processed |
| Legumes | 75 | 0 points = <18,75  1 point = 18,75-37,5  2 points = 37,5-75  3 points = >75 | Legumes  Soy |
| Nuts | 50 | 0 points = <12,5  1 point = 12,5-25  2 points = 25-50  3 points = >50 | Nuts |
| Unsaturated oils | 40 | 0 points = <10  1 point = 10-20  2 points = 20-40  3 points = >40 | Fatty acids, monounsaturated  Fatty acids, polyunsaturated |
| Added sugar | 31 | 0 points = >124  1 point = 62-124  2 points = 31-62  3 points = <31 | Added sugar |

* Reference intake based on an energy intake of 2500 kcal (1).

**Criteria for cut-off score is based on a study by Stubbendorff et al. (17).

***Foods included for each component are found in the DCH study.

****Pork was not available as a separate variable, therefore the points in this component counts double.

**Supplementary table 2** HR and 95% CI for the association between adherence to the EAT-Lancet diet score and colorectal cancer by adherence to the EAT-Lancet diet stratified by sex

| HR for colorectal cancer | | Events, *n* | | Person-years | | Model 1a* | | | | Model 1b** | | | | Model 2*** | | | | Model 3**** | | | |
| --- | --- | --- | --- | --- | --- | --- | --- | --- | --- | --- | --- | --- | --- | --- | --- | --- | --- | --- | --- | --- | --- |
| EAT-Lancet diet score |  | |  | |  | |  | |  | |  | |  | |  | |  | |  | |  |
| Men n=26,561 |  | |  | | HR | | 95%CI | | HR | | 95%CI | | HR | | 95%CI | | HR | | 95%CI | |  |
| <16 points | 146 | | 61,538 | | Reference | | | | Reference | | | | Reference | | | | Reference | | | |  |
| 17-19 points | 396 | | 176,541 | | 0.93 | | [0.77, 1.13] | | 0.95 | | [0.79, 1.15] | | 0.96 | | [0.79, 1.16] | | 0.95 | | [0.79, 1.16] | |  |
| 20-21 points | 282 | | 137,949 | | 0.85 | | [0.70, 1.04] | | 0.89 | | [0.72, 1.09] | | 0.90 | | [0.73, 1.10] | | 0.90 | | [0.73, 1.10] | |  |
| 22-23 points | 153 | | 77,273 | | 0.82 | | [0.66, 1.03] | | 0.86 | | [0.68, 1.08] | | 0.88 | | [0.69, 1.10] | | 0.87 | | [0.69, 1.10] | |  |
| >24 points | 51 | | 30,580 | | 0.70 | | [0.51, 0.96] | | 0.75 | | [0.54, 1.04] | | 0.77 | | [0.56, 1.07] | | 0.77 | | [0.55, 1.06] | |  |
| Per 10 points | 1028 | | 483,882 | | 0.72 | | [0.57, 0.91] | | 0.78 | | [0.61, 0.99] | | 0.80 | | [0.63, 1.01] | | 0.79 | | [0.62, 1.00] | |  |
| Women n=29,090 | |  |  |  | |  | |  | |  | |  | |  | |  | |  | |  |  |
| <16 points | 58 | | 35,847 | | Reference | | | | Reference | | | | Reference | | | | Reference | | | |  |
| 17-19 points | 238 | | 153,788 | | 0.95 | | [0.72, 1.27] | | 0.97 | | [0.73, 1.30] | | 0.98 | | [0.73, 1.30] | | 0.98 | | [0.73, 1.30] | |  |
| 20-21 points | 267 | | 163,690 | | 1.01 | | [0.76, 1.35] | | 1.05 | | [0.78, 1.40] | | 1.05 | | [0.79, 1.41] | | 1.06 | | [0.79, 1.41] | |  |
| 22-23 points | 193 | | 125,111 | | 0.97 | | [0.72, 1.30] | | 1.01 | | [0.75, 1.36] | | 1.01 | | [0.75, 1.37] | | 1.01 | | [0.75, 1.36] | |  |
| >24 points | 93 | | 80,942 | | 0.72 | | [0.52, 1.00] | | 0.76 | | [0.55, 1.06] | | 0.77 | | [0.55, 1.08] | | 0.77 | | [0.55, 1.08] | |  |
| Per 10 points | 849 | | 559,380 | | 0.82 | | [0.64, 1.05] | | 0.86 | | [0.67, 1.11] | | 0.88 | | [0.68, 1.13] | | 0.87 | | [0.68, 1.12] | |  |

All analyses were stratified by entry period (tertials) in the Cox proportional hazard models.

*Model 1a: Adjusted for age and sex.
** Model 1b: model 1a + diabetes, smoking status, physical activity level, alcohol consumption, education, hypertension, and hypercholesterolemia.

*** Model 2: model 1b + BMI.

**** Model 3: model 2 + total energy intake.

**Supplementary table 3** HR and 95% CI for the association between adherence to the EAT-Lancet diet score and colorectal cancer by adherence to the EAT-Lancet diet stratified by diabetes

| HR for colorectal cancer | Events, *n* | | Person-years | | Model 1a* | | | | Model 1b** | | | | Model 2*** | | | | Model 3**** | | | |  |
| --- | --- | --- | --- | --- | --- | --- | --- | --- | --- | --- | --- | --- | --- | --- | --- | --- | --- | --- | --- | --- | --- |
| EAT-Lancet diet score |  | |  | |  | |  | |  | |  | |  | |  | |  | |  | |  |
| Participants with diabetes n=1168 |  | |  | | HR | | 95% CI | | HR | | 95% CI | | HR | | 95% CI | | HR | | 95% CI | | |
| Per 10 points | 51 | | 18,742 | | 0.54 | | [0.21, 1.41] | | 0.55 | | [0.20, 1.47] | | 0.56 | | [0.21, 1.52] | | 0.57 | | [0.21, 1.53] | |  |
| Participants without diabetes n=54,483 | |  |  |  | |  | |  | |  | |  | |  | |  | |  | |  |  |
| Per 10 points | 1826 | | 1,024,520 | | 0.77 | | [0.65, 0.91] | | 0.82 | | [0.69, 0.98] | | 0.84 | | [0.70, 1.00] | | 0.83 | | [0.70, 0.99] | |  |

All analyses were stratified by entry period (tertials) in the Cox proportional hazard models.

*Model 1a: Adjusted for age and sex.
** Model 1b: model 1a + diabetes, smoking status, physical activity level, alcohol consumption, education, hypertension, and hypercholesterolemia.

*** Model 2: model 1b + BMI.

**** Model 3: model 2 + total energy intake.

**Supplementary Table 4** HR and 95% CI for the association between adherence to the EAT-Lancet diet score and colorectal cancer, colon cancer and rectal cancer across quartiles in the four adjusted models

| HR for colorectal cancer | Events, *n* | Person-years | Model 1a* | | Model 1b** | | Model 2*** | | Model 3**** | |
| --- | --- | --- | --- | --- | --- | --- | --- | --- | --- | --- |
| EAT-Lancet diet score |  |  | HR | 95% CI | HR | 95% CI | HR | 95% CI | HR | 95% CI |
| <17 points | 591 | 289,183 | Reference | | Reference | | Reference | | Reference | |
| 18-19 points | 521 | 293,261 | 0.88 | [0.78, 0.99] | 0.90 | [0.80, 1.02] | 0.91 | [0.81, 1.02] | 0.91 | [0.81, 1.02] |
| 20-21 points | 481 | 267,022 | 0.93 | [0.82, 1.05] | 0.95 | [0.84, 1.08] | 0.96 | [0.85, 1.09] | 0.96 | [0.85, 1.09] |
| >22 points | 284 | 193,795 | 0.78 | [0.68, 0.90] | 0.82 | [0.70, 0.95] | 0.83 | [0.72, 0.96] | 0.83 | [0.71, 0.96] |
| HR for colon cancer |  |  |  | |  | |  | |  | |
| EAT-Lancet diet score |  |  |  |  |  |  |  |  |  |  |
| <17 points | 396 | 289,183 | Reference | | Reference | | Reference | | Reference | |
| 18-19 points | 344 | 293,261 | 0.86 | [0.74, 0.99] | 0.88 | [0.76, 1.02] | 0.89 | [0.77, 1.03] | 0.89 | [0.77, 1.03] |
| 20-21 points | 314 | 267,022 | 0.88 | [0.76, 1.03] | 0.91 | [0.78, 1.06] | 0.92 | [0.79, 1.07] | 0.92 | [0.79, 1.07] |
| >22 points | 200 | 193,795 | 0.79 | [0.67, 0.94] | 0.83 | [0.69, 0.99] | 0.84 | [0.70, 1.00] | 0.84 | [0.70, 1.00] |
| HR for rectal cancer |  |  |  | |  | |  | |  | |
| EAT-Lancet diet score |  |  |  |  |  |  |  |  |  |  |
| <17 points | 195 | 289,183 | Reference | | Reference | | Reference | | Reference | |
| 18-19 points | 177 | 293,261 | 0.93 | [0.76, 1.14] | 0.95 | [0.77, 1.16] | 0.95 | [0.78, 1.17] | 0.95 | [0.78, 1.17] |
| 20-21 points | 167 | 267,022 | 1.02 | [0.83, 1.26] | 1.04 | [0.84, 1.29] | 1.05 | [0.85, 1.30] | 1.05 | [0.85, 1.30] |
| >22 points | 84 | 193,795 | 0.76 | [0.59, 0.99] | 0.78 | [0.60, 1.02] | 0.80 | [0.61, 1.05] | 0.80 | [0.61, 1.04] |

All analyses were stratified by entry period (tertiles) in the Cox proportional hazard models.

*Model 1a: Adjusted for age and sex.
** Model 1b: model 1a + diabetes, smoking status, physical activity level, alcohol consumption, education, hypertension, and hypercholesterolemia.

*** Model 2: model 1b + BMI.

**** Model 3: model 2 + total energy intake.

**Supplementary table 5:** HR and 95% CI for the association between adherence to the EAT-Lancet diet score and colorectal cancer in model 2, four years after baseline

| HR for colorectal cancer | Events, *n* | Person-years | Model 2* | |
| --- | --- | --- | --- | --- |
| EAT-Lancet diet score |  |  | HR | 95% CI |
| Per 10 points | 1796 | 1,043,171 | 0.82 | [0.68, 0.98] |

All analyses were stratified by entry period (tertials) in the Cox proportional hazard models.

*Model 2: Adjusted for age, sex, diabetes, smoking status, physical activity level, alcohol consumption, education, hypertension, hypercholesterolemia, and BMI.

**Supplementary table 6.** HR and 95% CI for the association between adherence to the EAT-lancet diet score and colorectal cancer in people with diabetes and who answered ‘don't know’ if they have diabetes, in the four adjusted models.

| HR for colorectal cancer | Events, *n* | Person-years | Model 1a* | | Model 1b** | | Model 2*** | | Model 3**** | |
| --- | --- | --- | --- | --- | --- | --- | --- | --- | --- | --- |
| EAT-Lancet diet score |  |  | HR | 95% CI | HR | 95% CI | HR | 95% CI | HR | 95% CI |
| <16 points | 13 | 7,439 | Reference | | Reference | | Reference | | Reference | |
| 17-19 points | 57 | 20,460 | 1.64 | [0.90, 3.00] | 1.57 | [0.85, 2.88] | 1.57 | [0.85, 2.88] | 1.56 | [0.85, 2.87] |
| 20-21 points | 32 | 17,375 | 1.12 | [0.59, 2.15] | 1.04 | [0.54, 2.02] | 1.05 | [0.54, 2.03] | 1.05 | [0.54, 2.03] |
| 22-23 points | 30 | 12,795 | 1.40 | [0.72, 2.70] | 1.23 | [0.62, 2.41] | 1.25 | [0.63, 2.45] | 1.24 | [0.63, 2.45] |
| >24 points | 12 | 6,900 | 1.08 | [0.49, 2.40] | 0.95 | [0.42, 2.16] | 0.97 | [0.43, 2.20] | 0.96 | [0.42, 2.18] |
| Per 10 points | 144 | 64,972 | 0.92 | [0.52, 1.64] | 0.81 | [0.44, 1.49] | 0.83 | [0.45, 1.53] | 0.82 | [0.45, 1.52] |

All analyses were stratified by entry period (tertials) in the Cox proportional hazard models.

*Model 1a: Adjusted for age and sex.
** Model 1b: model 1a + diabetes, smoking status, physical activity level, alcohol consumption, education, hypertension, and hypercholesterolemia.

*** Model 2: model 1b + BMI.

**** Model 3: model 2 + total energy intake.
